# Executive cognitive tests for the evaluation of patients with Parkinson’s disease

**DOI:** 10.1590/S1980-57642009DN20300008

**Published:** 2008

**Authors:** Emmanuelle Silva Tavares Sobreira, Marina Ceres Silva Pena, José Humberto Silva Filho, Carolina Pinto Souza, Guiomar Nascimento Oliveira, Vitor Tumas, Francisco de Assis Carvalho do Vale

**Affiliations:** 1 Psychologist, Investigator of the Group of Behavioral Neurology (GNC) of the University Hospital, Faculty of Medicine of Ribeirão Preto, University of São Paulo (HCFMRP-USP), Master’s Student enrolled on the Postgraduate Program in Mental Health, Faculty of Medicine of Ribeirão Preto, University of São Paulo(PGSM-FMRP-USP); 2 Psychologist, Doctor of Psychology, Professor of the Department of Psychology, Federal University of Amazonas (UFAM), Investigator of the Group of Behavioral Neurology (GNC) of the University Hospital, Faculty of Medicine of Ribeirão Preto, University of São Paulo (HCFMRP-USP).; 3 Neurologist of the University Hospital, Faculty of Medicine of Ribeirão Preto, University of São Paulo (HCFMRP-USP).; 4 Neurologist, Professor Doctor, Faculty of Medicine of Ribeirão Preto, University of São Paulo (FMRP-USP), Coordinator of the Outpatient Clinic of Movement Disorders, University Hospital, Faculty of Medicine of Ribeirão Preto, University of São Paulo (HCFMRP-USP).; 5 Neurologist, Coordinator of the Group of Behavioral Neurology (GNC), University Hospital, Faculty of Medicine of Ribeirão Preto, University of São Paulo (HCFMRP-USP).

**Keywords:** Parkinson’s disease, executive functions, cognitive tests

## Abstract

Parkinson’s disease (PD) is characterized by changes in movement, which are later followed by cognitive, behavioral and psychological changes. The objective of the present study was to correlate different tests used to examine executive functions in PD patients followed at a specialized outpatient clinic.

**Methods:**

Thirty-five patients with idiopathic PD aged 63.0 years on average and with mean schooling of 5.5±4.2 years, were examined using the following tests: *Mattis Dementia Rating Scale* (MDRS), *Scales for Outcomes of Parkinson’s Disease-Cognition* (SCOPA-COG), *Wisconsin Card Sorting Test* (WCST), *Frontal Assessment Battery* (FAB), *Digit Span – Inverse Order* (IO) (a subtest of the WAIS III) and *Verbal Fluency Test* (category animals).

**Results:**

Significant correlations were detected between FAB and MDRS Conceptualization (0.814), MDRS Initiation/Perseveration (I/P) and SCOPA-COG Executive Function (0.643), FAB and MDRS I/P (0.601), FAB and Verbal Fluency (0.602), MDRS I/P and MDRS Conceptualization (0.558), Verbal Fluency and MDRS I/P (0.529), MDRS Attention and SCOPA-COG Executive Function (0.495), MDRS Conceptualization and SCOPA-COG Executive Function (0.520), FAB and Digit Span (OI) (0.503), Verbal Fluency and MDRS Conceptualization (0.501), and WCST perseverative errors and FAB (–0.379), WCST perseverative errors and MDRS Conceptualization (0.445), WCST perseverative errors and MDRS I/P (–0.407) and WCST categories completed and MDRS Conceptualization (0.382).

**Discussion:**

The results demonstrated strong correlations between most of the tests applied, but no associations were detected between the WCST and the other tests, a fact that may be explained by the heterogeneity of scores obtained in the tests by the patients evaluated. A difficulty of the present study was the lack of a control groups for the establishment of adequate standards for this population.

## Abstract (pt)

A doença de Parkinson (DP) caracteriza-se por alterações do movimento, cognitivas, comportamentais e psicológicas. O objetivo do presente estudo foi correlacionar diferentes testes que examinam funções executivas em pacientes com DP acompanhados em um ambulatório especializado.

**Métodos:**

Foram avaliados 35 pacientes com DP idiopática com idade média de 63,0 anos e escolaridade média de 5,5±4,2 utilizando os seguintes testes: *Escala de Avaliação de demência de Mattis* (EADM), *Escala de Desfechos da Doença de Parkinso*n (SCOPA-COG), *Teste Wisconsin de Classificação de Cartas* (WCST), *Bateria de Avaliação Frontal* (BAF), *Amplitude de Dígitos-Ordem Inversa* (OI) (subteste do WAIS III) e *Teste de Fluência Verbal* (categoria animais).

**Resultados:**

Foram encontradas correlações significativas entre BAF e EADM Conceituação (0,814), EADM Iniciativa/Perseveração (I/P) e SCOPA-COG Funções Executivas (0,643), BAF e EADM I/P (0,601), BAF e Fluência Verbal (0,602) e EADM I/P e EADM Conceituação (0,558), Fluência Verbal e EADM I/P (0,529), EADM Atenção e SCOPA-COG Funções Executivas (0,49), EADM Conceituação e SCOPA-COG Funções Executivas (0,495), BAF e Amplitude de dígitos (OI) (0,503), Fluência Verbal e EADM Conceituação (0,501), WCST Erros Perseverativos e BAF (–0,379), WCST erros perseverativos e MDRS Conceptualization (–0,445), WCST erros perseverativos e EADM I/P (–0,407) e WCST categorias completas e EADM Conceituação (0,382).

**Discussão:**

Os resultados evidenciaram fortes correlações entre a maioria dos testes aplicados. Uma dificuldade do estudo presente diz respeito à falta de grupo controle para estabelecer padrões adequados a essa população.

Parkinson’s disease (PD) is a progressive neurological disorder characterized by motor disturbances and ranks the second most common neurodegenerative disease. PD affects 1 to 2% of individuals older than 60 years and 4 to 5% of individuals older than 85 years and corresponds to about 75% of all forms of parkinsonism.^1-6^

PD is associated with a wide gamut of cognitive, psychological and behavioral symptoms (psychosis and depression, among others) that significantly impair the quality of life of affected individuals, while also having an impact on the life of the caregiver.^1,7,8^ It is known that about 80% of patients develop cognitive changes detectable by clinical evaluation during the course of the disease, including cases of dementia. Some cognitive deficits such as those affecting executive and visuospatial function and memory occur during the early phases of the disease.

Executive function are among the most frequently described cognitive changes in patients with PD (30%).^1,7^ These functions refer to principles of cognitive organization and mental processes involved in the changing situations of daily life. They are adaptive skills since they modify mental strategies in response to environmental contingencies in order to reach an efficient and adequate solution for these situations.^9^

Executive functions are skills that require planning and execution of activities, including task initiation, attention, concentration, selectivity of stimuli, abstraction ability, planning, flexibility, mental control, self-control, working memory, and impulse inhibition.^9^

In view of the above considerations, the aim of the present study was to evaluate the performance of patients with PD in cognitive tests that explore executive functions, and to examine the correlations between complex and more simple bedside cognitive tests.

## Methods

### Patients and procedures

We evaluated consecutive patients with PD that attended an outpatient Movement Disorders Clinic at the Ribeirão Preto School of Medicine Hospital. Inclusion criteria were diagnosis of PD^10^ and possibility to return at a later date to complete the cognitive evaluation. Patients without schooling were included if they were not illiterate and could read and write. Exclusion criteria were: presence of psychotic symptoms, score on the 15-item Geriatric Depression Scale suggestive of depression^11^ and the use of antidepressive, anxiolytic and/or anticonvulsant drugs.

The clinical assessment of the patients was conducted in two stages. First, the patient was evaluated by a neurologist, who applied the Unified PD Rating Scale (UPDRS), Hoehn & Yahr and Schwab & England scales, Mini Mental State Examination, Survey of Subjective Memory Complaints, Frontal Assessment Battery, Verbal Fluency, Digit Span (DO and IO), Word List, Clock Drawing Task (CLOX 1 and 2), Interlocking Finger Test, Pfeffer Questionnaire and Clinical Dementia Rating. Patients were reevaluated at a later date, with a maximum interval of 30 days from the first evaluation. Patients were was assessed by a psychologist who applied the Wisconsin Card Sorting Test, Dementia Rating Scale, Hooper Visual Organization Test, Judgment of Line Orientation-Form V, Scale of Outcomes of Parkinson Disease-Cognitive and the Neuropsychiatry Inventory. The first evaluations were performed in around 45-60 minutes, while the second evaluations were performed in 90–120 minutes.

For clinical evaluation we used a shortened version of the UPDRS motor subscale containing only 8 items. This shortened version scored the same signs evaluated by the Short Parkinson’s Evaluation Scale^12^ but used the original 5-point items of the UPDRS. This scale was proven to have good reliability and validity in Brazilian patients with PD.^13^

This study was approved by the local ethics committee and patients included in this study gave their informed consent to participate.

### Description of the tests

The following tests and subscales were used for the study:

#### Mattis Dementia Rating Scale – MDRS

A scale used to evaluate cognitive status in general, extensively used as an auxiliary instrument for the diagnosis of dementias. The scale evaluates attention, initiative, perseverance, construction, conceptualization, and memory. It is used to detect losses of memory, language, orientation, attention, and praxis domains. In addition, it is a test that has been standardized and validated in Brazil. For the present study we used the data obtained on the *Attention, Initiation/Perseveration and Conceptualization* subscales as a specific battery of executive functions.^14^

#### Scale of Outcomes of Parkinson Disease – SCOPA-COG

A scale used to evaluate cognitive function in patients with PD by measuring the following domains: memory and learning, attention, executive functions, visuospatial function, and memory. We used a validated Brazilian version of the scale.^15^ The *Attention and Executive Function* subscales were used as a specific battery to test the executive functions.^16^

#### Wisconsin Card Sorting Test (WCST)

A test entailing the sorting of cards according to color, shape or number (short form, 64 cards), with the main objective of identifying signs of cerebral dysfunction and determining cognitive performance for problem solving. The measures obtained by the test concern executive functions, a set of competences that requires strategic planning, organized exploration based on environmental feedback in order to modify cognitive contexts, and to direct behavior toward goals with self-control, and modulate impulsive responses. The test has been standardized and validated in Brazil.^17^

#### Frontal Assessment Battery (FAB)

A test of semantic fluency: animal category. The FAB is a simple scale for the assessment of frontal functions which has not yet been validated in Brazil. We used a translated version of the original scale. It consists of 6 items that examine conceptualization (recognizing similarities), mental flexibility, motor programming, sensitivity to interference, inhibitory control, and environmental autonomy. The mental flexibility test examines verbal fluency and in the present study semantic fluency was also tested (names of animals within 1 minute).^18^

#### Digit Span – Inverse Order (IO)(a subtest of the WAIS III)

The objective of this instrument is to assess attention and working memory.^19^

#### Verbal Fluency Test (category animals)

Assesses executive functions, semantic memory and language. The test was performed in one minute. In this period, the patients state all the animal names they can remember. The cutoff is 9 for children and illiterate patients, and 13 for patients who have studied for more than 8 years.^20^

### Statistical analysis

The variables were processed using a database and statistical analysis carried out using the SPSS 13.0 software. Firstly, all variables were analyzed from a descriptive viewpoint, with calculation of means, standard deviations, and minimum/maximum values of the quantitative data. For categorical variables, relative and absolute frequencies were calculated. Non-parametric testing was used because variables did not present a normal distribution. Spearman’s correlation coefficient was used to study the correlations between cognitive tests, where the levels of significance used for the test were 5% and 1% (p<0.05, 95% or p<0.01, 99% confidence interval).

## Results

The clinical, demographic characteristics and results of the cognitive tests of the patients evaluated are presented in Table 1. The correlations between the cognitive tests are presented in Table 2

**Table 1:** Clinical and demographic characteristics, and results of cognitive tests in PD patients.

Variables	Mean±SD (Range)
Patients assessed	35
Gender (men:women)	21:14
Age	63.1±12.4 years (34 to 87 years)
Age at PD presentation	57.1±12.5 years (34 to 83 years)
Duration of disease	7.0±4.3 years (2 to 19 years)
Education (years)	5.5±4.1 years (0 to 19 years)
Hoehn and Yahr	2±0.6 (0 to 3)
Schwab and England	81%±17 (50 to 90)
Motor UPDRS (shortened version)	11.7±6.6 (4 to 26)
MDRS	123.4±12.7 (92 to 141)
MDRS Attention	34.31±1.9 (27 to 37)
MDRS Initiation/Perseveration	31.84±5.3 (16 to 37)
MDRS Conceptualization	29.9±5.0 (20 to 38)
SCOPA-COG	18.4±6.2 (5 to 28)
SCOPA-COG Attention	2.7±1.4 (0 to 4)
SCOPA-COG Executive Function	6.6±2.8 (2 to 12)
FAB	10.1±3.7 (1 to 16)
Verbal Fluency	10.7±3.8 (4 to 23)
Digit Span (IO)	3.0±0.9 (2 to 5)
WCST categories completed	1.2±1.1 (0 to 3)
WCST perseverative errors	23.6±13.2(5 to 46)
WCST non-perseverative errors	7.8±4.9(0 to 22)
MMSE	24.8±3.0 (18 to 29)

MDRS, Mattis Dementia Rating; SCOPA-COG, Scale, Scales of Outcomes of Parkinson Disease; FAB, Frontal Assessment Battery. Verbal Fluency (category animals). Digit Span inverse order (subtest of WAIS III); WCST, Wisconsin Card Sorting Test; MMSE, Mini Mental State Examination; UPDRS, Unified Parkinson's Disease Rating Scale; Hoehn and Yahr Staging of Parkinson's Disease; Schwab and England Activities of Daily Living; SD, standard deviation.

**Table 2:** Correlations between cognitive tests (Spearman correlation coefficient (r_s_)).

	FAB	MDRS Conceptualization	MDRS Initiation/ Perseveration	SC Exec Function	WCST perseverative errors	WCST categories completed
FAB	-	0.814*(p=0.001)	0.601* (p=0.001)	-	-0.379† (p=0.039)	-
MDRS conceptualization	0.814* (p=0.001)	-	0.558* (p=0.001)	0.520* (p=0.002)	-0.445* (p=0.010)	0.382† (p=0.028)
MDRS initiation/perseveration	0.603* (p=0.001)	0.558* (p=0.001)	-	0.643* (p=0.001)	-0.407† (p=0.019)	-
MDRS attention	0.364† (p=0.041)	-	-	0.495* (p=0.003)	-	-
SC executive function	0.541* (p=0.002)	0.520* (p=0.002)	0.643* (p=0.001)	-	-	-
WCST perseverative errors	-0.379† (p=0.039)	-0.445* (p=0.010)	-0.407† (p=0.019)	-	-	-
Verbal fluency	0.602* (p=0.001)	0.501* (p=0.003)	0.529* (p=0.002)	0.371† (p=0.040)	-0.577* (p=0.001)	-
Digit span (IO)	0.503* (p=0.003)	-	0.358† (p=0.044)	0.507* (p=0.004)	-	-

FAB, Frontal Assessment Battery; MDRS, Mattis Dementia Rating Scale; SC, SCOPA-COG - Scales of Outcomes of Parkinson Disease; WCST, Wisconsin Card Sorting Test; Digit Span - Inverse Order (subtest of WAIS III); Verbal Fluency (category animals);

* Correlations significant at the 0.01 level (2-tailed);

† Correlation significant at the 0.05 level (2-tailed)

We found no correlation between duration of the disease, the shortened score of motor UPDRS, Hoehn and Yahr stage, Schwab and England functional capacity and the cognitive tests. The total scores on the MMSE, SCOPA-COG and MDRS were highly correlated. The FAB and verbal fluency test scores were also correlated well with most of the specific executive tests, followed by the SCOPA-COG and finally the MMSE

## Discussion

The patients evaluated presented low mean scores on the following tests: MDRS, Conceptualization, SCOPA-COG Executive Function, FAB, Digit Span (IO), WCST perseverative errors and WSCT non-perseverative errors. These findings may be explained by the fact that cognitive impairment occurs frequently in PD, and is more prominent in the memory and executive function domains.^16^ However, it is important to point out that we cannot rule out the presence of cultural and schooling effects on the results of these tests. A limitation in the interpretation of the present study was the lack of adequate standards of performance in these tests for the Brazilian population. Most scales had not been adapted, validated or standardized, indicating the need for further studies in this area.

We found no correlation between the performance on the cognitive tests and patient age, duration of the disease, severity of motor symptoms, stage of the disease and functional capacity. This finding was unexpected and can be explained by the small number of patients evaluated in this sample.

The FAB scale is easy to apply and significantly related to Wisconsin and MDRS scores. Significant correlations were also detected between the FAB and the remaining tests, with emphasis on the strong correlation between the FAB and the MDRS Conceptualization subscale. This was possibly due to the fact that both instruments assess executive functions, among other cognitive functions. However, in the present study the correlations between FAB and WCST perseverative errors, MDRS I/P and WCST perseverative errors, WCST perseverative errors and MDRS Conceptualization and WCST categories completed and MDRS Conceptualization were moderate, perhaps owing to the specificity of the population studied.^18,21^ The verbal fluency test was also well correlated with other more specific cognitive tests. Our findings suggest that the FAB and verbal fluency test may be valuable tools for assessing executive functions in patients with PD.

It is also important to point out that the correlations between the subscales of the MDRS and the SCOPA-COG were mostly moderate. In contrast, MDRS I/P and SCOPA-COG Executive Function were strongly correlated, perhaps because the MDRS scale more specifically assesses the executive functions, as is also the case for the SCOPA-COG subscale. It is interesting to note that the correlation between the SCOPA-COG Attention and MDRS Attention subscales was non-significant, probably because, although they apparently measure the same function, they measure different aspects of attention.

Although the WCST is described in the literature as one of the best tests for assessing of executive functions, it did not correlate in the present study with the remaining tests that also assess the same cognitive function, suggesting independence between them. This may be explained by the heterogeneity of scoring on the tests by the patients evaluated.

Cognitive symptoms are a frequent complaint of patients with PD, mainly manifesting in terms of executive function deficits. Their effect on daily life may be profound, yet few studies and tests specifically evaluating this impairment in patients with PD are available, indicating the need for further study in this area.^7,22,23^
